# Alexithymia and psychopathological symptoms in adolescent outpatients and mothers suffering from migraines: a case control study

**DOI:** 10.1186/s10194-016-0640-y

**Published:** 2016-04-19

**Authors:** Rita Cerutti, Carmela Valastro, Samuela Tarantino, Massimiliano Valeriani, Noemi Faedda, Valentina Spensieri, Vincenzo Guidetti

**Affiliations:** Department of Dynamic and Clinical Psychology, Sapienza University of Rome, Via degli Apuli, 1, Rome, Italy; Headeache Center, Division of Neurology, Ospedale Pediatrico Bambino Gesù, IRCCS, Piazza S.Onofrio, 4, Rome, Italy; Department of Pediatrics and Child and Adolescent Neuropsychiatry, Sapienza University of Rome, Via dei Sabelli, 108-00185 Rome, Italy

**Keywords:** Alexithymia, Migraine, Psychopathological risk, Adolescents, Mothers

## Abstract

**Background:**

Headache is a common disorder affecting a growing number of children and adolescents. In recent years, there has been an increase in scientific interest in exploring the relationship between migraine and emotional regulation, and in particular, the impact of emotional dysregulation on mental and physical health. The present study aims to explore the relationship between migraine and alexithymia among adolescents and their mothers as well as the impact of this association on mental health. An additional aim is to verify whether alexithymia may be a predictor of psychopathological symptoms in adolescents and mothers with migraines.

**Methods:**

A total of 212 subjects were involved in this study. The sample was divided into (a) Experimental Group (EG) consisting of 106 subjects (53 adolescents and 53 mothers) with a diagnosis of migraine according to International Classification of Headache Disorders (ICHD-3) and (b) Control Group (CG) including 106 subjects (53 adolescents and 53 mothers) without a diagnosis of migraine. All participants completed the Toronto Alexithymia Scale to assess alexithymia and the Symptom Checklist-90-R to assess psychopathological symptoms.

**Results:**

Higher rates of alexithymia were found in the adolescents and mothers of the EG in comparison to the adolescents and mothers of the CG. Furthermore, adolescents and mothers experiencing both migraine and alexithymia, demonstrated a higher risk of psychopathology.

**Conclusions:**

Findings from this study provide evidence that the co-occurrence of migraine and alexithymia increases the risk of psychopathology for both adolescents and their mothers.

## Background

Headache is one of the most common complaints within the pediatric population, with a prevalence rate ranging from 5 % to 25 % of children and adolescents reporting suffering from severe or frequent headaches according to the International Classification of Headache Disorders criteria [1]. Research has documented that gender has an influence on the characteristics of the headache and its impact varies across age groups [2]. Specifically, some studies have highlighted that migraine affects males and females equally at a young age (<14 years). Conversely during adolescence (>14 years) and young adulthood, and in correspondence with the reproductive years, it is more common in girls [3] suggesting that hormones or hormonal changes are somehow linked to migraines [4, 5]. The increased number of changes (e.g. biological, cognitive, and social) during the transition to adolescence can increase negative affect, emotional reactivity, and risk for internalizing symptoms [6], with gender differences being evident. In particular, female adolescents are more likely to suffer from anxiety and depression and it has been observed that these conditions can aggravate or precede headaches (e.g. migraines) [7].

Headaches could have a negative impact on the daily life and activities of young people, including lower ratings of quality of life, poorer physical and mental health, and more missed school days. With regard to comorbid psychopathology in children and adolescents suffering from primary headaches (migraine or tension type headache), there is still controversy since some studies have shown that it is tension type headache (TTH) that are influential on psychopathological symptoms [8], while others indicate the opposite, particularly for internalizing disorders [9]. The findings of a recent study [10] add evidence for a significant difference in the internalizing problems of children who suffer from migraine in comparison to those with TTH. Specifically, it was noted, that migraine was typically accompanied by disability, loss of performance [11], higher rates of psychiatric disorders, including anxiety, depression, [9, 12] suicidal thoughts and suicide attempts [7, 13], as well as emotional and behavioral problems [14]. Furthermore, studies have found that children and adolescents with migraine experience difficulty in expressing emotional states [8, 15]. In addition, there is evidence that children with recurrent pain disorders (i.e., migraine, tension type headache, abdominal pain, skeletal muscle pain) show a higher level of alexithymic features compared with healthy controls [16–19]. Unlike studies among adult populations, there is little research on the association between recurring episodic neurological disorders, such as primary headaches, and alexithymia in adolescence [20]. Alexithymia refers to a personality construct that includes a reduced ability to identify and describe feelings, a limited imagination, and a concrete, externally-oriented way of thinking. Alexithymia has been recognized as associated with several somatic illnesses and mental disorders [21]. Furthemore studies detected that the presence of alexithymia has a negative influence on the course of symptoms in psychiatric disorders [22]. In particular some authors have suggested that alexithymia could be considered a vulnerability factor in the development of psychiatric disorders such as depression [22–24].

Theoretical and research contributions have shown how the ability to identify and express emotions plays a key role in the construction of health and illness [25] as well as demonstrated the significant association between physical symptoms and difficulty in identifying and describing feelings [26]. Previous studies have highlighted how the development of emotion regulation can be impaired by inadequate parenting, having an impact on the development of alexithymia [27].

Since there is some evidence that early childhood experiences influence the development and ability to manage emotions appropriately, it could be hypothesized that the quality of the parent–child relationship plays a role in the intergenerational transmission of alexithymia. Indeed, some studies have highlighted a significant association between parent and child alexithymia scores [28, 29]. In particular, low maternal care seems to be correlated with alexithymic features, especially with the difficulty to describe feelings [30].

Moreover, other studies on monozygotic and dizygotic twin pairs have found that among alexithymic features, externally-oriented thinking was associated with genetic factors while difficulty identifying and describing emotions were influenced by environmental factors [31].

The findings of recent studies on adolescent populations have demonstrated a relationship between alexithymic traits and various mental disorders [32]. Specifically, Honkalampi and colleagues [33] found a link between alexithymia and depressive symptoms in a large sample (*n* = 7,087) of Finnish adolescents.

Although a number of research studies have investigated the relationship between primary headaches, alexithymia and the risk of developing psychiatric comorbidities in adults [34], the number of studies on adolescents and the above associations is quite limited [16, 20].

In light of previous findings, we hypothesize higher alexithymia scores in both adolescents and mothers suffering from migraines compared with adolescents and mothers without headache. Furthermore, we expect to find a higher level of risk for psychopathological symptomatology (e.g., anxiety and depression symptoms) in participants with migraines in contrast to those without migraines.

The aims of this study are: (a) to assess the relationship between migraine diagnosis according to ICHD-3 criteria and alexithymia; (b) to explore the association between the co-occurrence of migraine and alexithymic features and the risk of psychopathology; c) to test the hypothesis that alexithymia may predict psychopathological symptoms in adolescents and mothers with migraines, and d) to determine the differences between adolescents and their mothers suffering from migraine versus adolescents and mothers in the control (non-headache) groups.

## Methods

### Participants

A total of 212 subjects were enrolled in this study. Participants were divided into four distinct groups (a) Adolescent Experimental Group (AEG) vs (b) Mother Experimental Group (MEG) vs (c) Adolescent Control Group (ACG) vs (d) Mother Control Group (MCG).

### Experimental group

53 adolescents outpatients (23 males, 43.4 % and 30 females, 56.6 %; mean age = 13.39; SD = 2.39) and 53 mothers (mean age = 41.77; SD = 3.92) attending the Headache Center of the Department of Paediatrics and Child and Adolescent Neuropsychiatry, Sapienza, University of Rome and the Headeache Center of the Bambino Gesù Children’s Hospital, located in Rome, participated in this study.

Inclusion criteria included a migraine diagnosis at least 6 months previously, without pharmacological prophylaxis. Migraine was diagnosed according to the ICHD-3 beta criteria [35].

### Control group

53 adolescents (25 male, 47.2 % and 28 female, 52.8 %; mean age =12.51; SD = 0.61) and 53 mothers (MCG; mean age = 41.35; SD = 5.31) were recruited from a secondary public school in an urban area in central Italy and were involved in this study as part of a health promotion project. Inclusion criteria involved no medical history of headaches or medical/organic diseases. Parents completed a schedule that assessed their age, sex, race/ethnicity and education level as well as health status of their child, answering if the child was under pharmacological and/or psychological therapy or whether he/she already had a diagnosed infection or other medical illness (e.g., diabetes, epilepsy, loss of consciousness, asthma/allergies, heart condition, lung condition, hearing impairment or specifying whether other medical conditions were present at the moment of the study). The two groups did not significantly differ with respect to age, gender and size.

### Ethical aspects

Participation was obtained through a written informed consent procedure from both adolescents and mothers. The study was approved by the Ethics Committee of the Medicine and Psychology Faculty, Sapienza University of Rome.

### Measures

#### Headache diagnosis

A headache diagnosis was conducted with the *Headache Questionnaire* used to investigate the presence or absence of migraine according to the ICHD-3 beta criteria [35]. The questionnaire detects the pain characteristics, frequency and period of headache attacks, and associated symptomatology.

#### Alexithymia

Alexithymia was assessed by the *Toronto Alexithymia Scale-20* item version (TAS-20) [36, 37]. The TAS-20 is a 20 item self-report scale, the most widely used for assessing alexithymia in both research and clinical practice. Responses to each item are made on a 5-point Likert scale ranging from 1 (strongly disagree) to 5 (strongly agree). Factor analysis has consistently yielded three factors that assess the salient features of the alexithymia construct: difficulty identifying feelings (F1); difficulty describing feelings (F2); and externally-oriented thinking (F3).

F1 is an index of respondents’ difficulty in identifying an experience as an affective state: seven items [1, 3, 6, 7, 9, 13, 14] assess the ability to identify feelings and to distinguish them from the somatic sensation that accompanies emotional arousal.

F2 consists of five items [2, 4, 11, 12, 17] that assess the capacity to name and describe feelings to other people. F3 consists of eight items [5, 8, 10, 15, 16, 18–20] assessing externally-oriented thinking. To assess the prevalence of alexithymia, the TAS-20 scores were categorized according to the suggested cut off: ≥ 61 indicating alexithymia; 51 to 60 possible alexithymia (borderline range) < 51 indicating no alexithymia. In the present study, reliability (internal consistency, Cronbach alpha) on the whole sample for the total score was 0.80, and 0.76, 0.56 and 0.62 for F1, F2 and F3, respectively.

#### Psychological distress

*The Symptom Checklist*-*90*-*R* (SCL-90-R) [38] was used to assess psychological distress. This scale is a 90-item self-report symptom inventory used by clinical psychologists, psychiatrists and mental health professionals in medical and educational settings as well as for research purposes. Responses to each item are rated on a five point scale of distress ranging from 0 (not at all) to 4 (extremely). The SCL-90-R is intended to measure symptom severity of nine different subscales (somatization, obsessive-compulsive, interpersonal sensitivity, depression, anxiety, hostility, phobic anxiety, paranoid ideation and psychoticism). Scores on the nine symptom dimensions are expressed as a profile of symptoms. There are three suggested global indices of distress that value the level and severity of pathology: the Global Severity Index (GSI), the Positive Symptom Distress Index (PSDI), and the Positive Symptom Total (PST).

The scale measures symptomatology within the past week. Internal consistency of the nine scales as calculated on the present sample was: 0.86 for somatization, 0.77 for obsessive-compulsive symptoms, 0.78 for interpersonal sensitivity, 0.87 for depression, 0.80 for anxiety, 0.69 for hostility, 0.74 for phobic anxiety, 0.76 for paranoid ideation and 0.77 for psychoticism.

### Statistical analyses

SPSS 19.0 (Statistical Package for Social Sciences) was used for statistical analyses. Descriptive statistical analyses were used to investigate participants' characteristics. Chi-square was used to compare experimental groups (adolescents and mothers) to control groups (adolescents and mothers). A variation analysis (ANOVA) was performed for the three Factors and the total score of the TAS-20 as well as for the nine dimensions and the total score of the SCL-90-R. Regression analysis will be employed to test the hypothesis that alexithymia may predict psychopathological symptoms in adolescents and mothers with migraine. In all analyses, a significance level was set at alpha 0.05, two-tailed.

## Results

### Alexithymia

A frequency analysis was performed to verify the distribution of the frequencies of alexithymia within the groups (EG vs CG). As observed in Table 1, the prevalence of alexithymia was more frequent among adolescents with migraine than in the adolescent control group, as expected. This difference was statistically significant (Chi-Square 7.966 df 2 *p* < 0.05). Similarly, the frequency of alexithymia was also higher among mothers with migraine in comparison to the mother control group, although this difference was not statistically significant (Chi-Square 1.861 df 2 *p* = .394) (Table 1). Linear regression analysis was conducted with the SCL 90-R total score as the dependent variable and the TAS-20 total score as the predictor. Findings revealed a significant prediction of psychopathology by alexithymia score in both adolescents and mothers with migraine (adolescents: β = .362, *p* = .008, R^2^ = .131; mothers: β = .524, *p* = .000, R^2^ = .260). Furthermore, the SCL 90-R total score and the SCL 90-R subscales were analyzed as a function of alexithymic migraineurs. Adolescents above the TAS-20 cut off (*n* = 17) reported higher mean scores on the SCL 90-R total score and on the following subscales: somatization, obsessive-compulsive symptoms, interpersonal sensitivity, depression, anxiety, paranoid ideation and psychoticism (Table 2). Mothers above the TAS-20 cut off (*n* = 20) reported higher mean scores on the SCL 90-R total score and on all of the SCL 90-R subscales (Table 2).

**Table 1:** Frequency of Alexithymia in Adolescent and Mother Groups

	Total (AEG)	Total (MEG)	Total (ACG)	Total (MCG)
	n (%)	n (%)	n (%)	n (%)
No Alexithymia (<51)	18 (34)	23 (43.4)	32 (60.4)	30 (56.6)
Borderline Range (51 to 60)	18 (34)	10 (18.9)	13 (24.5)	8 (15.1)
Alexithymia (>61)	17 (32.1)	20 (37.7)	8 (15.1)	15 (28.3)

*AEG* Adolescent experimental group, *MEG* Mother experimental group, *ACG* Adolescent control group, *MCG* Mother control group

**Table 2:** Means and standard deviations of migraineurs above or under the TAS-20 cut off

Measures	TAS 20 (>61)	TAS 20 (<51)	TAS 20 (>61)	TAS 20 (<−51)
	AEG (*n* = 17)	AEG (*n* = 36)	MEG (*n* = 20)	MEG (*n* = 33)
	Mean (SD)	Mean (SD)	Mean (SD)	Mean (SD)
SCL 90-R_tot	95.76* (51.3)	56.08 (42.7)	94.65* (44.2)	52.33 (38.6)
Somatization	13.82* (9.5)	8.05 (6.2)	15.85* (7.7)	10.39 (8.1)
Obsessive-compulsive	12.23* (7.3)	7.33 (5.3)	12.75* (4.1)	7.72 (6.2)
Interpersonal Sensitivity	10.18* (6.3)	5.9 (4.9)	8.15* (4.7)	3.64 (3.2)
Depression	15.50* (9.9)	9.38 (8.6)	15.55* (9.0)	8.76 (6.9)
Anxiety	12.23* (6.9)	6.77 (4.9)	10.90^*^(6.3)	6.42 (4.4)
Hostility	6.18 (5.1)	3.75 (3.9)	4.25* (2.9)	2.33 (1.9)
Phobic-Anxiety	3.23 (3.5)	2.33 (2.6)	7.25* (4.9)	2.91 (3.4)
Paranoid Ideation	7.88* (5.3)	3.86 (3.4)	4.55* (3.5)	2.28 (2.8)
Psychoticism	7.18* (4.9)	3.72 (5.2)	8.10* (5.2)	3.64 (3.9)

*p < .05; *AEG* Adolescent Experimental Group, *MEG* Mother Experimental Group

An analysis of variance (ANOVA) was conducted to compare the mean scores on the TAS-20 completed by the two groups of adolescents (AEG vs ACG). As shown in Table 3 the AEG demonstrated higher mean scores than the ACG and a statistically significant difference (*p* <0.01 and *p* <0.05) was found for the three factors (F1, F2, F3) as well as for the total score of the TAS-20. However, no statistically significant gender differences were observed (*p* > .10).

**Table 3:** Means for the TAS-20 in Adolescent and Mother Groups

	Total(AEG)	Total(ACG)	F	P value*	Total(MEG)	Total(MCG)	F	*P* value*
	M (SD)	M (SD)			M (SD)	M (SD)		
F1	16.41 (6.88)	12.84 (4.85)	9.494	<0.01	16.09 (5.52)	13.45 (5.78)	5.782	<0.05
F2	13.94 (3.69)	11.90 (3.35)	8.835	<0.05	12.75 (4.03)	12.28 (4.61)	.314	.577
F3	25.94 (5.49)	23.45 (5.68)	5.254	<0.01	26.41 (5.55)	25.13 (7.00)	1.092	.298
TAS-20	56.30 (12.14)	48.20 (10.30)	13.694	<0.01	55.26 (12.54)	50.86 (14.53)	2.779	.099

*P value: Comparison between experimental and control groups. *AEG* Adolescent experimental group, *ACG* Adolescent control group, *F* Fisher’s F ratio, *MEG* Mother experimental group, *MCG* Mother control group, *F1* difficulty identifying feelings, *F2* difficulty describing feelings, *F3* externally-oriented thinking, *TAS*-*20* TAS-20 total score, *M* mean, *SD* standard deviation

With respect to the two groups of mothers (MEG vs MCG), the analysis of variance between the mean scores highlighted higher scores in the MEG compared to the MCG. However, results evidenced a statistically significant difference (*p* < 0.05) only on the F1 factor of the TAS-20 (Table 3).

### Psychopathological symptoms

As showed in Table 4 and in line with our hypothesis, the means of the AEG were higher than the means of the ACG for both the nine dimensions and the total score of the SCL-90-R. A statistically significant difference for “*somatization*” (*p* < 0,01), “*depression*” (*p* < 0,01), “*anxiety*” (*p* < 0.01) and the SCL-90-R total score (*p* < 0.05) was found.

**Table 4:** Means for the SCL-90-R in Adolescent and Mother Groups

	Total(AEG)	Total(ACG)	F	P value*	Total(MEG)	Total(MCG)	F	*P* value*
	M (SD)	M (SD)			M (SD)	M (SD)		
Somatization	9.90 (7.85)	6.30 (6.01)	7.035	<0.01	12.45 (8.35)	7.84 (6.89)	9.576	<0.01
Obsessive-Compulsive	8.90 (6.36)	7.18 (6.25)	1.963	.164	9.62 (5.99)	5.86 (5.68)	10.943	<0.01
Interpersonal Sensitivity	7.32 (5.74)	6.16 (5.02)	1.204	.275	5.33 (4.42)	4.37 (5.14)	1.065	.304
Depression	11.37 (9.43)	7.35 (5.72)	7.029	<0.01	11.32 (8.36)	7.50 (7.97)	5.765	<0.05
Anxiety	8.52 (6.15)	5.43 (4.53)	8.674	<0.01	8.11 (5.60)	6.09 (6.48)	2.941	.089
Hostility	4.52 (4.45)	3.79 (3.37)	.919	.340	3.05 (2.45)	2.66 (2.98)	.557	.457
Phobic Anxiety	2.62 (2.93)	2.50 (3.04)	.038	.846	4.54 (4.55)	1.41 (2.47)	19.339	<0.01
Paranoid Ideation	5.15 (4.50)	4.58 (3.83)	.485	.488	3.13 (3.25)	3.33 (3.79)	.091	.763
Psychoticism	4.83 (5.35)	4.07 (5.03)	.559	.456	5.32 (4.89)	3.32 (4.36)	4.928	<0.05
SCL-90-R Total Score	68.81 (48.87)	50.98 (37.19)	4.467	<0.05	68.32 (45.39)	46.30 (41.99)	6.718	<0.05

^*^P value: Comparison between experimental and control groups. *AEG* Adolescent experimental group, *ACG* Adolescent control group, *F* Fisher’s F ratio, *MEG* Mother experimental group, *MCG* Mother control group, *M* mean, *SD* standard deviation

With regards to gender differences, females reported higher mean scores than males on all of the SCL-90-R dimensions with the exception of the “*phobic anxiety*” dimension in which males presented a higher mean score than females. However, no statistical differences were observed between females and males in either the AE or AC groups (*p* > .10).

The MEG showed higher mean scores on the SCL-90-R dimensions than did the MCG, with the exception of the “*paranoid dimension*”, wherein the MCG reported higher means than the MEG, but this result was not statistically different. Instead, statistically significant differences were found for mean scores on the following dimensions: “*somatization*” (*p* < 0.01), “*obsessive*-*compulsive*” (*p* < 0.01), “*depression*” (*p* < 0.05), “*anxiety*” (*p* < 0.01), “*phobic anxiety*” (*p* < 0.05), “*psychoticism*” (*p* < 0.05) and for the SCL-90-R total score (*p* < 0.05) (Table 4).

### The relationship between migraine, alexithymia and psychopathological symptoms

In order to assess the relationship between migraine, alexithymia and psychological distress in the EGs versus the CGs, a comparison of mean scores on the SCL-90-R reported by our sample, in the presence or absence of alexithymia and with or without migraine was carried out. As presented in Table 5, the mean scores obtained by the experimental groups (AG and MG) as well as the control groups (AC and MG) on the SCL-90-R total score were higher in participants with alexithymia than participants without alexithymia. However, adolescents and mothers with migraine and alexithymia reported higher scores on the SCL-90-R than adolescents and mothers without migraine, highlighting a more severe risk of psychopathology risk.

**Table 5:** Mean scores on the SCL-90-R and the TAS-20 of the Experimental versus Control Groups

	SCL-90-R (AEG)	SCL-90-R (ACG)	SCL-90-R (MEG)	SCL-90-R (MCG)
	M (SD)	M (SD)	M (SD)	M (SD)
No Alexithymia	47.94 (41.93)	40.50 (31.33)	47.43 (38.36)	33.06 (29.74)
Alexithymia	95.76 (51.34)	83.25 (46.14)	94.65 (44.22)	76.13 (46.09)

*AEG* Adolescent experimental group, *ACG* Adolescent control group, *MEG* Mother experimental group, *MCG* Mother control group, *M* mean, *SD* standard deviation

## Discussion

Higher rates of alexithymia were found in adolescents and mothers of the EGs than adolescents and mothers of the CGs. Previous research studies on headache patients have highlighted their higher level of alexithymia, as well as their increased prevalence of depression, anxiety, behavioral symptoms and somatic complaints [39, 40].

Empirical evidence of the important role of the family in pediatric patients with primary headaches has grown significantly in the last few years [41, 42]. Parents with deficits in the ability to regulate emotions and to express feelings can inhibit the ability of their children to self-regulate their emotional states, influencing their bodily experience with the tendency to somatization [43]. The results of this study confirm our expectations highlighting that adolescents and mothers with a migraine diagnosis demonstrate a higher level of alexithymia compared to the CGs, with a statistically significant difference for the adolescent group of migraine sufferers (Chi-Square 7.966 df 2 *p* < 0.05). This finding is consistent with the study by Gatta and colleagues [16], that showed higher levels of alexithymic features in children and mothers with primary headaches, compared with healthy controls. Moreover, previous studies on adults have shown how the ability to identify and express emotions plays a key role in the construction of health and illness [25] and highlighted the association between physical symptoms and difficulty in identifying and describing feelings [26]. Findings from past studies found a significant relationship between alexithymia and TTH [34, 44], but not between alexithymia and migraine. Focusing on a migraine diagnosis, our data provide evidence that mothers with migraine have higher levels of alexithymia compared to mothers in the control group, showing a heightened difficulty in identifying feelings.

Additionally, our findings are consistent with results that have emphasized the presence of alexithymia in mothers of children with somatic complaints and the influence of maternal psychological symptoms, maternal chronic pain, and parenting stress on children’s somatic complaints [45]. Vieira and colleagues [40] found that women with migraine had higher levels of depression, anxiety and alexithymia, and lower levels of quality of life, self-reflection and insight, compared to controls. Few studies have examined the relationship between alexithymia and migraine in children and adolescents; the most recent work on the topic of primary headaches acknowledged how children with recurring pain disorders (e.g., migraine, tension type headache) showed higher levels of alexithymia than their peers without such disorders [16]. Our data demonstrated a statistically significant difference (*p* <0.01 and *p* <0.05) for the three factors of the alexithymia construct (difficulty identifying feelings, F1; difficulty describing feelings, F2; externally-oriented thinking, F3) as well as for the total score of the TAS-20. These findings suggest an association between migraine and alexithymia and are consistent with those obtained in a previous study investigating the relationship between alexithymia and TTH [16]. In particular, the third factor of the TAS-20 (externally-oriented thinking) seems to largely characterize the adolescents and mothers of the experimental group rather than those of the control group which are distinguished mainly by a difficulty in identifying and describing feelings (F1 and F2). This result lends support to common psychological and behavioral characteristics (e.g., competition, perfectionism, ambition, rigidity and tendency to suppress emotions) described in previous studies including youth [46] and adults with migraines [47]. It could be argued that the high scores on externally-oriented thinking reported by the mothers in both the experimental and control groups (i.e., a poor imagination and thinking that is strongly anchored to the external reality rather than to introspection) may affect the ability of children in identifying and describing feelings. This result seems to confirm what has been previously observed in children with migraine revealing a rigorous and strict familiar environment, demanding in discipline with high levels of conventionality, low levels of intimacy and emotional participation [48]. A previous study by Valera and Berenbaum [31] explored the possible intergenerational transmission of alexithymic traits suggesting that externally-oriented thinking was influenced by genetic factors. The authors studied 45 monozygotic and 32 same-gender dizygotic twin pairs and found that the “external-oriented thinking” factor was associated with genetic factors, whereas the “difficulty identifying emotions” and “difficulty describing emotions” were influenced by shared environmental factors.

Focusing on comorbid psychopathology, findings from our study demonstrate that both adolescents and mothers suffering from migraine, also appear to experience greater psychological distress than the adolescents and mothers in the control groups. In particular, adolescents with migraine report significantly higher scores than the control group adolescents on somatization, depression and anxiety dimensions of the SCL-90-R. Whereas mothers with migraine report significantly higher scores than the control group mothers on somatization, depression, phobic anxiety, obsessive-compulsive and psychoticism dimensions of the SCL-90-R. These results are consistent with previous studies that have shown a correlation between headache and internalizing symptoms (e.g., anxiety and depression) [10] and other studies that found alexithymia to be associated in children, adolescents and adults with depression and anxiety [32, 33, 43], somatization, reduced empathy and an impaired ability to recognize the emotions of others, psychoticism and phobia [28]. Previous studies detected a neurochemical pathways shared by migraine, psychopathology and emotional regulation, that could play a role in this association. Considering the relevance of genetic factors in both migraine and affective disorders, the hypothesis of an involvement of a common mechanism has been postulated to explain the implication of similar neurochemical abnormalities [13, 49, 50]. Breslau and colleagues [51] found significant bidirectional relationships between major depression and migraine: migraine seems to predict first-onset depression and depression seems to predict first-onset migraine. Different factors may be involved in the co-occurrence of these two disorders, such as genetic-neurodevelopmental relationships [52]. Noteworthy and differently from results of a recent study [44] that showed no differences in alexithymic scores of mothers with or without headache, our data suggest that mothers of headache patients report higher levels of alexithymia compared to other mothers. The correlation within migraine patients, for both adolescents and mothers with alexithymic features, points to a possible intergenerational transmission of alexithymia. A child’s alexithymia may reflect the mother’s deficit in regulating emotions and expressing feelings. The ability to understand ones’ own and others’ emotions and to display emotions, contributes to the achievement of both intrapersonal (e.g., individual well-being) and interpersonal (e.g., maintenance of important social relationships) well-being. There is converging evidence that youths’ abilities in emotional regulation are shaped by their environment. There are a lot of variables implicated in the development of emotional regulation, including family, school, peer, and genetic factors. However, parents represent one of the most influential sources by which adolescents learn to label, identify, and interpret emotions [53, 54]. Finally, data from this study support our hypothesis that in patients with migraine, alexithymia was a significant predictor of self-reported psychopathological symptoms. It is useful to acknowledge the following limitations of this study. First, our clinical sample comprises a specific population of patients with migraine attending a third-level center and may reflect referral bias. Second, we used self-report questionnaires to gather data (TAS-20 and SCL-90-R). Third, this study involved a relatively small number of participants. Furthermore, caution should be considered in drawing conclusions from this cross-sectional study. Future longitudinal research is needed to investigate the relationship between the variables considered in this study. In summary, this research confirmed the notion that adolescent alexithymic characteristics represent a risk factor for both mental and somatic disorders, on the assumption that difficulty in relating can have different outcomes depending on its interaction with other factors [25, 55].

## Conclusion

The findings of this study provide important information about the relationship between levels of alexithymia in children and mothers with a migraine diagnosis and suggest that the co-occurrence of migraine and alexithymia increases the risk of psychopathology for both adolescents and their mothers. The association between primary headaches, particularly migraine, and alexithymia should be further investigated in prospective studies to understand how the presence of a deficit in parental emotional regulation affects the severity and frequency of a migraine disorder in children. Indeed, it is of the utmost importance for treatment efficacy to take into account the deficits in cognitive processing and emotional regulation of alexithymic patients especially when alexithymia is present in conjunction with other disorders. The knowledge of the relationship between somatic symptoms, such as primary headaches, alexithymia and psychological problems, can provide important clinical information for a preventive, targeted and efficacious treatment program.

## Abbreviations

ACG: Adolescent Control Group
AEG: Adolescent Experimental Group
CG: Control Group
EG: Experimental Group
F1: Difficulty Identifying Feelings
F2: Difficulty Describing Feelings
F3: Externally-Oriented Thinking
GSI: Global Severity Index
ICHD-3: International Classification of Headache Disorders
MCG: Mother Control Group
MEG: Mother Experimental Group
PSDI: Positive Symptom Distress Index
PST: Positive Symptom Total
SCL-90-R: Symptom Checklist-90-Revised
TAS-20: Toronto Alexithymia Scale
TTH: Tension Type Headache
